# Can precancerous stem cells be risk markers for malignant transformation in the oral mucosa?

**DOI:** 10.1186/s11658-023-00441-0

**Published:** 2023-04-07

**Authors:** Shan Wang, Liu Ying, Shu-Yi Yu, Jie Bai, Chunbo Hao

**Affiliations:** 1 Department of Oral Pathology, School of Stomatology, Hainan Medical University, Haikou, 571199 People’s Republic of China; 2 Department of Stomatology, The Second Affiliated Hospital of Hainan Medical University, Haikou, 570216 People’s Republic of China; 3 College of Pharmacy, Hainan Medical University, Haikou, 571199 People’s Republic of China; 4 Pharmacy Department, First Affiliated Hospital of Jiamusi University, Jiamusi, 154003 People’s Republic of China; 5 Department of Ophthalmology, the Fourth Affiliated Hospital, Zhejiang University School of Medicine, Yiwu, 322000 People’s Republic of China; 6 Department of Stomatology, Hainan General Hospital (Hainan Affiliated Hospital of Hainan Medical University), Haikou, 570100 People’s Republic of China

**Keywords:** Precancerous stem cells, Cancer stem cells, Oral cancer, Dysplasia, Malignant transformation

## Abstract

Accurate assessment of the carcinogenic potential of oral mucosal diseases can significantly reduce the prevalence of oral cancer. We speculate that precancerous stem cells (pCSCs) arise during the evolution of carcinomas based on long-term experimental findings, published literature, and the cancer stem cell (CSC) theory, wherein pCSCs exist in precancerous lesions and have characteristics of both CSCs and normal stem cells. This apparently contradictory feature may be the foundation of the reversible transformation of precancerous lesions. Predicting malignant transformation in potentially malignant oral illnesses would allow for focused treatment, prognosis, and secondary prevention. Currently available clinical assays for chromosomal instability and DNA aneuploidy have several deficiencies. We hope that our study will increase attention to pCSC research and lead to the development of novel strategies for the prevention and treatment of oral cancer by identifying pCSC markers.

**Graphical Abstract:**

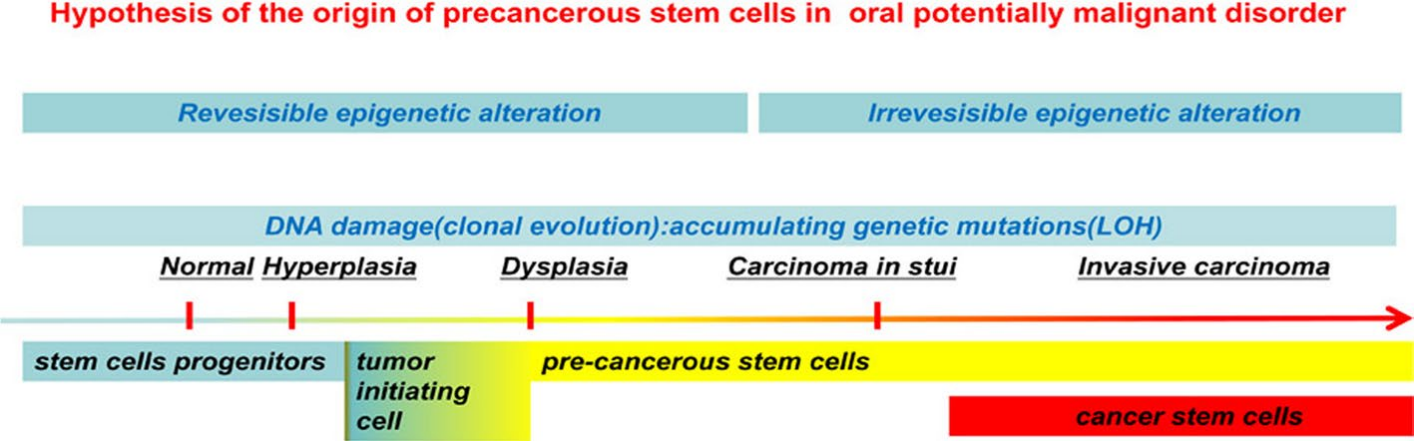

**Supplementary Information:**

The online version contains supplementary material available at 10.1186/s11658-023-00441-0.

## Introduction

Oral cancer is a common oral and maxillofacial cancer that accounts for approximately 3% of all malignant tumors [1]. Although the incidence is lower than that of other malignant tumors, it is often accompanied by local invasion and distant metastasis during diagnosis and treatment, and the overall 5-year survival rate is only 50–55% [2]. The cure rate of patients with focal or regional invasion is < 30%, which is poor given the improvements in cancer treatment. The survival rate is difficult to improve, mainly because oral cancer is often not diagnosed at an early stage [2–4].

Presently, the likelihood of an oral potentially malignant disorder (OPMD) progressing to oral cancer is determined according to histopathological findings, which are susceptible to inadequate and/or subjective qualitative interpretations [5]. Clinical assays for DNA ploidy and loss of heterozygosity (LOH) have a strong biological foundation and a predictive value that is unaffected by dysplasia grades or clinical characteristics [6]. Because of discrepancies in the expression of results and their application to populations with varying risk potentials, DNA ploidy and LOH assays cannot be directly compared. Moreover, malignant transformation cannot be accurately predicted in individual patients using either technique [6, 7].

DNA is damaged by both endogenous and exogenous sources, since it is not a static molecule but rather a dynamic moiety that interacts with a variety of chemical and physical variables. DNA damage changes its structure, which can lead to a genetic mutation if it is not repaired. Errors arising in stem and progenitor cells have a much greater influence on the tissue in which they are found than errors arising in postmitotic differentiated cells. Therefore, investigations on testing and maintaining the integrity of genomic DNA within precancerous stem cells (pCSCs), which are within the framework of the cancer stem cell (CSC) hypothesis, are necessary.

## Carcinogenesis can be explained by CSCs and pCSCs

Recently, the CSC hypothesis has received increased attention [8–11]. According to the hypothesis, only a small group of stem cell-like tumor cells can form new tumors. These cells are the original precursors of various cells in a tumor, whereas other intratumoral cells are limited in terms of their potential for proliferation and pluripotent differentiation. Evidence supports the idea that only a few cell subtypes within tumors can form tumors de novo during cancer evolution [12, 13]. Cells require three to six genetic mutations to become carcinogenic, and these accumulate over time [14]. In humans, buccal epithelial cells turn over 7–10 days [15]. Most epithelial cells have a short lifespan and cannot accumulate the genetic mutations necessary for oral squamous cell carcinoma (OSCC) to develop [16, 17]. Persistent stem cells in the oral epithelium of adults are the only cells that can accumulate sufficient mutations for OSCC to develop [18, 19]. In addition, normal adult stem cells (ASCs) self-renew, proliferate infinitely, have a multidirectional differentiation potential, similar to that of tumor cells, and can transform more easily into tumor stem cells than non-stem cells [20, 21]. Consequently, ASCs are considered the origin of cancer, rather than normal mature tissue cells.

**Fig. 1:**
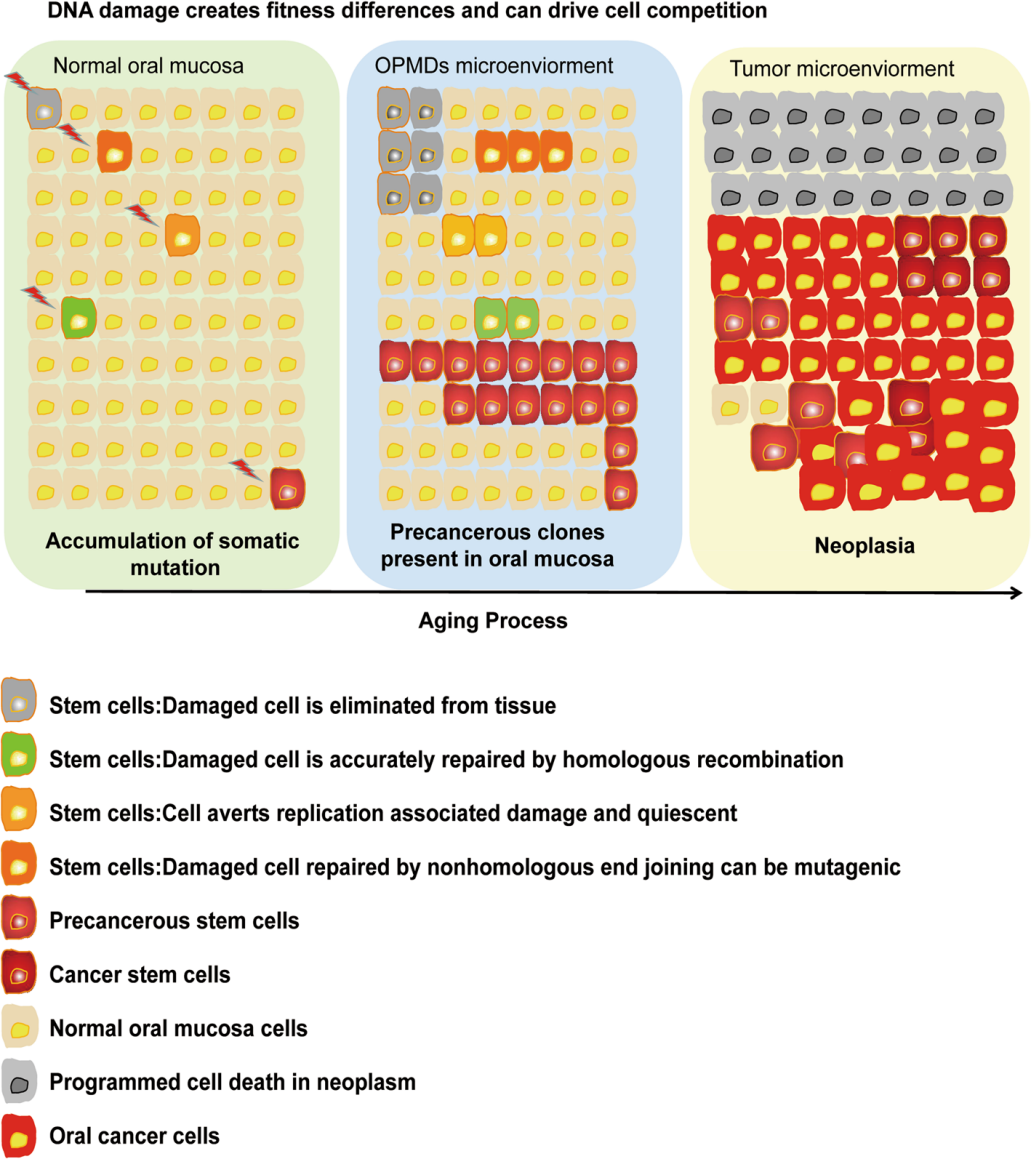
Mutations accumulate in young stem cells of normal oral mucosa (lawn green). Mosaic patches result from age-dependent clonal proliferation of mutant stem cells via positive selection or neutral drift. The precancerous stem cells (pCSCs) may appear, and their persistence may lead to cancer initiation (turquoise). Clonal proliferation of cancer driver genes may lead to cancer stem cell (CSC) and cancer initiation (wheat). The core concept of the above hypothesis is referred to as the somatic mosaicism with age theory [7]

Gao et al. discovered a new type of tumor cell, the aforementioned pCSCs, in a mouse lymphoma that had the characteristics of early CSCs, but had a similar clinical origin to that of precancerous lesions and the features of benign and malignant differentiation [22–27]. Min et al. distinguished the cellular behaviors and characteristics of metaplastic and dysplastic organoids derived from the Mist1-Kras (G12D) mouse stomach corpus. They believed that dysplastic stem cells might contribute to the cellular heterogeneity of dysplastic cell lineages [27] (Fig. 1). Thus, pCSCs are a stem cell type with potentially special characteristics. pCSCs have the characteristics of both NSCs and CSCs [26], and this is necessary in the early stage of CSC development. The discovery of pCSCs explains the variable phenotype and the ability of CSCs to differentiate and form tumors. Both pCSCs and CSCs are likely to function as stromal components of tumors, such as vasculogenic stem cells or progenitors. Thus, the current histopathological interpretation of the CSC hypothesis is that communication occurs during the process of conversion from a precancerous lesion to cancer (e.g., simple dysplasia to moderate dysplasia to carcinoma in situ).

The precancerous state is an important stage of cancer development because extensive heterogeneous changes can still be reversed [28–31]. This state must be regulated by pCSCs rather than CSCs, as pCSCs have the potential for promotion (malignant transformation) or regression (benign differentiation) [26]. The study of pCSCs in precancerous lesions is critical for prevention and early diagnosis of cancer. Zouabi et al. indicates that the occurrence of somatic mosaicism in aging may explain the existence of pCSCs [7]. Stem cells in precancerous clones have different fates under stress, such as elimination, repair, quiescence, or evolution into pCSCs. Thus, competition between stem cells for niche occupancy may impact the transition from a premalignant to a malignant state (Fig. 1).

What distinguishes pCSCs from CSCs? Various cancers have a prolonged precancerous stage that is histologically distinguishable from the cancerous stage [28, 29]. Although pCSCs might be more plastic than CSCs, CSCs transform from pCSCs with little phenotypic difference according to contemporary research, and all human CSC markers may be expressed on pCSCs [32]. Few mature pCSC and CSC lines have been derived from tumors and characterized. Moreover, distinguishing human pCSCs from CSCs using tumor reconstruction is difficult in animal models because human tumor xenografts are not histocompatible with lethally irradiated bone marrow-reconstituted (BMR) and immunocompetent (IC) mice [26], which is a problem because BMR mice can be used to assess pCSCs’ ability to differentiate benignly and self-renew, whereas IC mice can be used to determine whether the cells tested are pCSCs or CSCs. However, precancerous tissues grow orthotopically rather than heterotopically after transplantation [33], which may indicate that, compared with CSCs, pCSCs require distinct growth niches [26]. Therefore, Gao et al. distinguished human pCSCs from CSCs by their suitability for orthotopic or heterotopic xenotransplantation [26]. Additionally, Min et al. reported that they identified two different stem cell populations from Kras-induced dysplastic lineages of mouse stomach corpus, according to whether they survive and propagate in vivo [27].

## Current hypothesis and evidence of pCSCs in OPMDs

### Hypothesis of the origin of pCSCs in OPMDs

Although pCSCs are regarded as CSC precursors, their origin remains unclear. Gao et al. suggested that the origin of pCSCs could be ASCs, progenitors, or supplementary proliferating cells, such as epithelial cell precursors [26]. Multiple genetic changes are necessary for cell transformation, and an adequate cell cycle process is necessary for mutations induced by accumulated DNA damage to occur [22, 23]. ASCs are persistent cells that rarely enter the cell cycle, whereas progenitor cells might be target cells for the accumulation of genetic variations [33–35]. The aforementioned hypothesis is further supported by 4NQO-oral carcinogenesis mouse models, which have been used to demonstrate that the basal layer contains long-lived stem cells that produce progeny by asymmetric division to maintain homeostasis [36]. The progenitor cells may be most susceptible to oncogenic mutations, as tumor-initiating cells gradually obtain stem cell-like properties and consequently develop into pCSCs. Even so, ASCs or tissue-uncommitted stem cells might be potential sources of pCSCs [26, 32, 37].

### Implications of gene instability of pCSCs in OPMDs

The ability of several biomarkers to predict malignant transformation in OPMDs has been assessed [38–40]. A substantial amount of evidence obtained from follow-up, prospective, and therapeutic research supports chromosomal instability (CIN) and aneuploidy [41–43]. The molecular characteristics of pCSCs and CSCs are attributed to the abnormal activation or inhibition of oncogenes caused by gene instability. CIN and microsatellite instability (MIN) are common forms of gene instability in the pathogenesis of colon and oral cancers [44–47]. CIN can irreversibly result in aneuploidy in cancer cells, whereas cells with MIN are frequently diploid in the precancerous stage [48].

Amplifications and deletions are balanced out to generate diploid DNA, but chromosomally aneuploid cells, but no DNA measurement system can quantitatively distinguish normal diploid cells from aneuploid cells. This may be a clinical problem, since near-diploid aneuploidization may be an early stage in the development of OPMDs [49]. This might also explain how a DNA aneuploidy precursor can lead to diploid cancer. While other body sites have diploid carcinomas [50], whether oral carcinomas or their precursor OPMDs can be diploid cannot be concluded currently using DNA aneuploidy techniques [6].

**Fig. 2:**
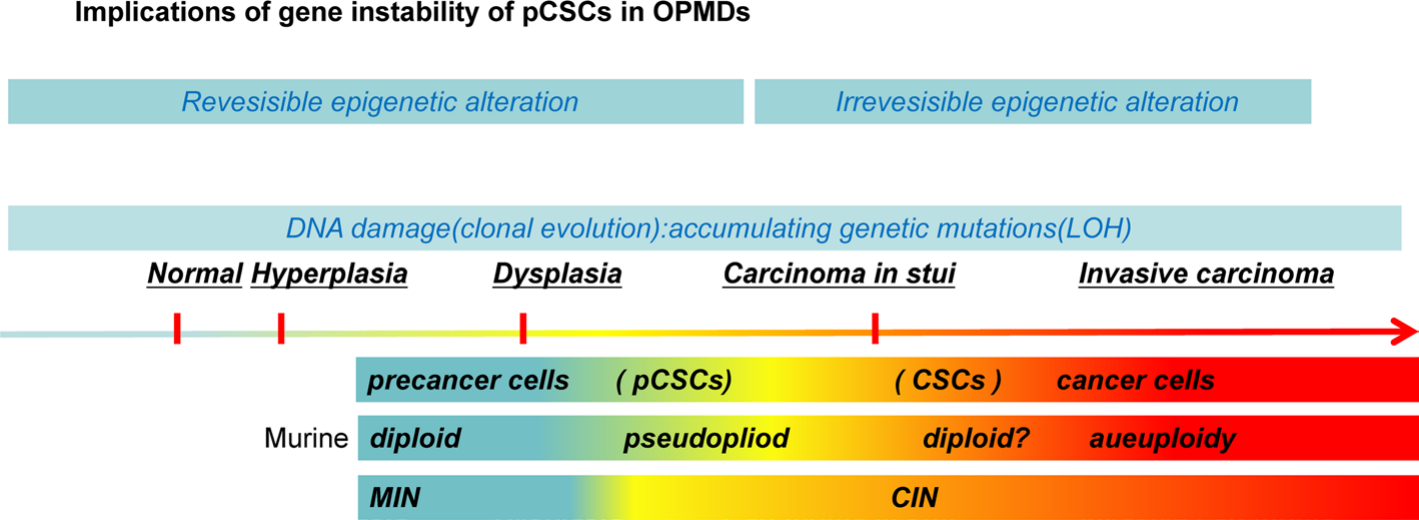
In precancerous stem cells (pCSCs), a qualitative mutation of oncogenes or tumor suppressor genes may result in the loss of the benign differentiation and commitment capability of cancer stem cells (CSCs). Murine pCSC clones are pseudodiploid, whereas CSC clones are usually diploid. The number of chromosomal translocations in CSCs does not increase compared with that in pCSCs. Cells with microsatellite instability (MIN) are normally approximately diploid. The core concept of the above hypothesis has been described elsewhere [26]. *LOH* loss of heterozygosity, *CIN* chromosomal instability

Murine pCSC clones are pseudodiploid with numerous chromosomal translocations, whereas mouse cancer cells are frequently aneuploid [26]. Although CSC clones are usually diploid, the number of chromosomal translocations does not increase compared with that in pCSCs, indicating that the malignant nature of cancer cells in certain tumors may be a result of the accumulation of qualitative rather than quantitative changes in genomic variation [26] (Fig. 2). The above assumption is more reasonable for multilayered squamous epithelia in oral carcinogenesis. Zaini et al. also showed that, on a cellular level, oral dysplastic lesions may contain only a few aneuploid cells, substantial copy number gain is uncommon, and changes may be caused by massive chromosomal fragment duplications. For loci with copy number gain, single stem lines (mainly pCSCs) are rather uniform, although gene amplification reveals a subclonal structure in some lesions [51].

Predicting precancerous stem cell markers could be a potential test or treatment, along with aneuploidy tests, for creating DNA measuring systems that can distinguish normal diploid cells from aneuploid cells.

### Effect of the immune microenvironment on pCSCs in OPMDs

Does the microenvironment promote or restrain the outgrowth of precancerous cells? It may be contradictory for cells with stem cell properties in the immune microenvironment [52]. Luan et al. used whole-genome sequencing on clonally expanded single liver stem cells cultured from patients with alcoholic cirrhosis, nonalcoholic steatohepatitis, and primary sclerosing cholangitis, which are precancerous liver disease conditions, and found no evidence of increased mutation accumulation or altered mutation types in the intrahepatic cholangiocyte organoids [53]. By contrast, an uninflamed liver micromilieu controls cell growth and CSC properties through oxidative phosphorylation in pancreatic ductal epithelial cells, which can induce metastasis of pancreatic cancer [54]. Objectively, whether OPMDs transform to OSCC depends on the interplay of different factors, including the cellular origin, molecular heterogeneity, and immunogenic potential associated with the microenvironment. For example. Chen et al. illustrated that potential precancerous lesions of colorectal cancer, such as serrated polyps, develop from differentiated cells through stomach metaplasia, whereas colorectal adenomas develop from WNT-driven stem cell growth. Prior to hypermutation, metaplasia-related damage is also linked to a cytotoxic immune microenvironment, driven in part by variations in antigen presentation associated with the status of tumor cell differentiation. Microsatellite unstable colorectal cancers contain distinct non-metaplastic regions where tumor cells acquire stem cell properties and cytotoxic immune cells are depleted [55]. We found that most OPMDs transformed to OSCC accompanied by inflammatory processes, and a challenging cytotoxic immune environment may be conducive for distinguishing pCSCs from ASCs or progenitor cells, or the further acquisition of pCSC characteristics by a minority of stem cells. Chen et al. found that the cytotoxic immune microenvironment is driven by mutant developed cells in lesions and not stem cells [55]. Another study using 4NQO carcinogenesis mouse models supported the conclusion, with an increase in the proportion of mesenchymal stem cells decreasing the proportion of T cells in precancerous tongues [56, 57]. Additionally, the antigen presentation machinery is inversely related to stemness in the human colon epithelium, which may partly underlie the differential stimulation of a cytotoxic immune response [55]. Data on the association of the immune microenvironment and precancerous stem cells in the oral epithelium should be sought in future studies.

### Potential markers expressed on pCSCs in OPMDs

pCSCs with characteristics including self-renewal, pluripotency, and multipotency of embryonic stem (ES) cells, germ line stem (GS) cells, and ASCs can express ASC-related genes such as *Bmi-1*, *Notch-1*, *Smo, OcT-4*, *TDGF-1*, *REX1*, and *Piwil2* [26], which are related to ES cells [58]. The expression of ES cell-related genes in pCSCs is unstable, which might be associated with their high sensitivity. Precancerous stem cells can abundantly and stably express the ES cell-related *Piwil2* gene, indicating that Piwil2 could serve as a reliable marker of pCSCs. The expression of Piwil2 is stable in precancerous tissues of various human organs [21, 22, 25, 26]. The Piwil2 protein is a small RNA-binding protein that plays a key role in germ cell maintenance in the testis. Additionally, it is widely expressed in colon, breast, prostate, gastrointestinal, ovarian, soft tissue, and endometrial cancers, but not in normal somatic and stem cells [28, 29, 31, 44]. We also found that Piwil2 could predict the malignant transformation of OPMDs [59]. Novel large-scale mechanistic investigations and prospective clinical studies are needed to determine the prognostic role of Piwil2 in this cancer model system. Known markers of OPMDs include CD24, CD44, Nestin, Sox2, and Nanog [30, 32, 38–40, 60]. Nevertheless, these markers lack obvious characteristics to distinguish pCSCs and CSCs. The lack of proper animal models and representative cell lines has led to a scarcity of mechanical evidence for pCSCs in oral carcinogenesis, despite the identification of stem cell markers from oral dysplasia tissue. Dysplastic organoids from the 4NQO-induced carcinogenic tongue of mice may be a suitable model in future research [61]. Overall, data on pCSC markers are limited, and we lack a well-defined underlying theory.

### Hints from long-lived epithelial stem cells and their clonal progeny in OPMD and OSCC

Marta et al. used K14-CreER tam/Rosa26LacZ mice to trace the mutational profiles in clonal cell populations derived from single long-lived epithelial stem cells (LLESCs). These mice were treated with 4NQO simultaneously to create a murine OSCC model. Using this lineage tracing approach, they demonstrated that LacZ + stem cells and their progeny can be followed during the pre-neoplastic stages and the formation of OSCC. Cell adhesion and development were found to be recurrent functional categories in 83% of OSCC (LLESC), whereas cell adhesion was the only recurrent functional category in 50% of OPMDs (LLESC). Mutation of Celsr1, Celsr3 and Ddr1 in LLESC involves in orientation and cell polarization, suggesting that mutations in these genes may alter key signaling pathways contributing OPMDs to OSCC development. Celsr1 and Fat4, both atypical cadherins [62, 63], play an important role in controlling planar cell polarity, which can help distinguish between precancerous and cancerous stem cells. Even in OPMDs (LLESC), copy number alteration (CNA) events are interspersed across the mouse chromosomes; OSCC (LLESC) exhibited 100-fold more CNA events than OPMD (LLESC), and the vast majority of these were amplifications. In addition to the identification of CNA events in OPMDs (LLESC) [64], common patterns have been identified among OPMDs (LLESC).

## A hypothesis explaining the specific properties of pCSCs: embryogenesis, carcinogenesis, and transdifferentiation

Here, we illustrate the concept of pCSCs using innovative tumorigenesis theories. Liu suggested the life code theory [65]. They concluded that the 32-cell morula can be considered a multinucleated giant cell (or 64n syncytium) during early development, with the zona pellucida acting as a cell membrane and cleavage functioning as endomitosis. The increase in the nuclear-to-cytoplasmic ratio follows the decrease in cell size, activating a series of embryonic transcription factors that dedifferentiate the parental genome from the zygotic genome. This phase includes morphologic changes from a morula to a blastocyst and the formation of an inner cell mass that gives rise to new embryonic life. If the differentiation process continues until complete maturation, the organism will have a normal life. A well-differentiated tumor will arise if differentiation is prevented at any step along the continuum from primordial germ cells to embryonic development to fetal organ maturation [60] (Additional file 1: Fig. S1). This is an avant-garde theory attempting to explain the relationship between embryogenesis and carcinogenesis. Illustrating how pCSCs in oral carcinogenesis also express the ES and GS markers appears to be reasonable. Coincidentally, Piechowski also inferred trophoblastic and sexual phenotypes in carcinogenesis [66]. Those that allow for migration and immortality are simply added to the basic set of cell characteristics. Only stem-like cells acquire these properties de novo, as they are the only ones capable of transdifferentiation, owing to their phenotypic plasticity [63]. We showed that stem-like cells may be a mixture of pCSCs and CSCs. Yet, pCSCs may possess more sex-specific properties considering that Gao et al. [26] distinguished pCSCs from CSCs by their suitability for orthotopic or heterotopic xenotransplantation; it should be acknowledged that pCSCs do not have a migration capability. Only CSCs may be able to result in metastasis (Additional file 2: Fig. S2).

One example of a sex-specific property of pCSCs is that, similar to somatic stem cells that commonly and spontaneously express telomerase, they may exhibit a resurgence of the germinal phenotype to implement cell immortality. That is to say, they have a pressing requirement for efficient genome maintenance because they are dealing with genome flaws and instability, as well as constant multiplication. Certain genome-supporting activities that are found in germinal cells [67] are reexpressed in pCSCs. Furthermore, the expression of oncofetal biomarkers may have a collateral effect.

Malignant transdifferentiation is a unique type of cell reprogramming with a crucial role in the precancer-to-cancer transformation and appears to be a plausible explanation for the reappearance of trophoblastic and sexual characteristics. In the absence of malignant transdifferentiation, premalignant lesions either slumber or wither through senescence and apoptosis. The hypothesis also explains the stem cell competition for niche occupancy.

## Conclusions

We have clarified that the characteristics of pCSCs differ from those of NSCs and CSCs, and that pCSCs in oral mucosal precancerous lesions should be investigated to determine their evolutionary processes from new perspectives, such as the life code and transdifferentiation theories. In addition, we have reinterpreted markers in pCSCs on the basis of these perspectives. Meanwhile, classification schemes for human OPMDs should focus on intrinsic features of tumor cells, including bulk gene expression (CIN and LOH), MIN, and the immune microenvironment from the perspective of pCSCs. Future in-depth studies may determine the risk of oral cancer lesions more accurately and supplement histopathological findings to judge whether a lesion is precancerous. Further investigations can provide more detailed advice regarding the early diagnosis and prevention of oral cancer and a reference for the targeted treatment of precancerous stem cells before oral cancer progression.

## Supplementary Information

**Additional file 1: Figure S1.** A model that ties together the human life cycle and the origins of malignancies. The germ cell and somatic cell life cycles are both a component of the normal human life cycle. However, neoplasia is a result of the giant cell life cycle. The life cycle of germ cells: during gametogenesis, the oocyte size increases progressively, and fertilization causes an increase in the nuclear-to-cytoplasmic (N/C) ratio, which activates the embryonic program. A typical life code is defined as five successive cleavage divisions from a single-celled zygote to yield a 32-cell morula (or 64n multinucleated giant cell). An aged or damaged somatic cell experiences an identity transformation, including cytoskeletal modification, to become a tumor preinitiation cell, which then undergoes senescence, resulting in an increase in cell size. Exogenous pressure, such as immunological microenvironment stress, acts as the “sperm,” triggering “somatic embryogenesis” by endocycling, resulting in large polyploid cancer cells (4n/pn) with a high N/C ratio. An endocycling cell goes through endomitosis or self-renewal and eventually becomes a morula-like multinucleated polyploid large cancer cell. To achieve stability, multinucleated polyploid large cancer cells become cellularized and result in a variety of undifferentiated tumors, in which pCSCs and CSCs are screened out through nuclear reprogramming. Endoreplication results in an increase in ploidy, which is referred to as a neoplastic life code. The core concept of the above hypothesis has been described previously [65].

**Additional file 2: Figure S2.** After reprogramming of typically switched off sexual and trophoblastic master genes, a precancer cell becomes malignant. Malignant cells are a phenotypic mix of primordial precancer cells with sexual-like and trophoblastic-like transdifferentiations, similar to precancerous stem cells (pCSCs) and cancer stem cells (CSCs). A relevant collateral consequence could be the expression of oncofetal biomarkers. Pouf1/Otc4, TDGF1, Zfp42/REX1, and Sox2 in pCSCs are associated with germ stem cell (GS) markers and CD24, CD44, Nestin, Sox2, Notch1, and Nanog are associated with trophoblasts. The core concept of the above hypothesis has been described previously [66].

## Data Availability

Not applicable. **Ethics approval and consent to participate** Not applicable.

## Abbreviations

ASCs: Adult stem cells
BMR: Bone marrow-reconstituted
CIN: Chromosomal instability
CSC: Cancer stem cell
CNA: Copy number alteration
CT80: Cancer/testis antigen 80
ES: Embryonic stem cell
GS: Germ line stem cell
IC: Immunocompetent
LOH: Loss of heterozygosity
LLESCs: Long-lived epithelial stem cells
MIN: Microsatellite instability
NSCs: Normal adult stem cells
OPMDs: Oral potentially malignant disorders
OSCC: Oral squamous cell carcinoma
PCSCs: Precancerous stem cells
Piwil2: P-element-induced wimpy testis-like 2
TDGF-1: Teratocarcinoma-derived growth factor 1
