# Supplementary figures and images for: Can precancerous stem cells be risk markers for malignant transformation in the oral mucosa?
Source: Cell Mol Biol Lett. 2023 Apr 7;28:30. doi: 10.1186/s11658-023-00441-0 (PMC10080963; doi:10.1186/s11658-023-00441-0)

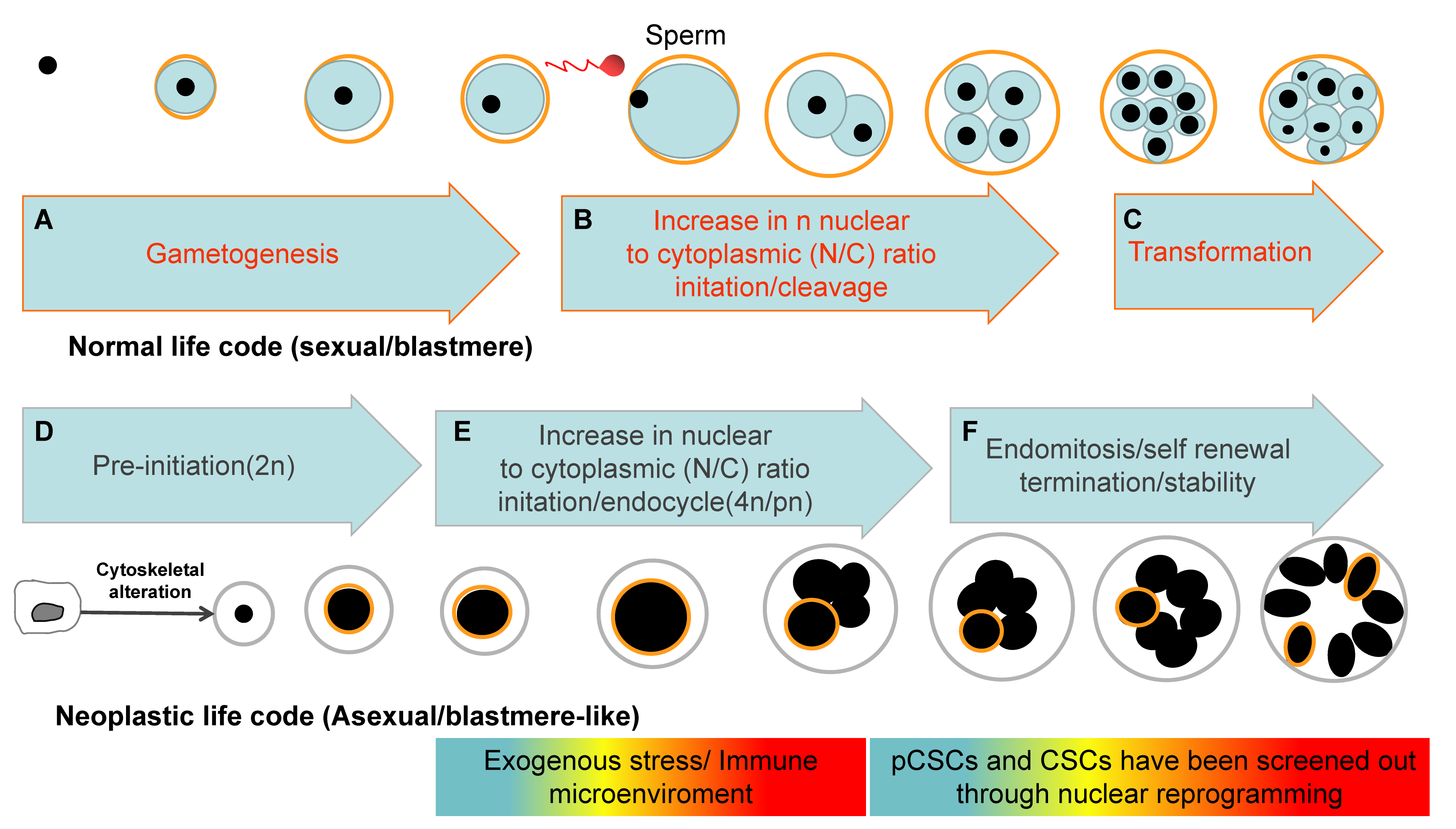

Supplement: Supplementary file 1 — Additional file 1: Figure S1. A model that ties together the human life cycle and the origins of malignancies. The germ cell and somatic cell life cycles are both a component of the normal human life cycle. However, neoplasia is a result of the giant cell life cycle. The life cycle of germ cells: during gametogenesis, the oocyte size increases progressively, and fertilization causes an increase in the nuclear-to-cytoplasmic (N/C) ratio, which activates the embryonic program. A typical life code is defined as five successive cleavage divisions from a single-celled zygote to yield a 32-cell morula (or 64n multinucleated giant cell). An aged or damaged somatic cell experiences an identity transformation, including cytoskeletal modification, to become a tumor preinitiation cell, which then undergoes senescence, resulting in an increase in cell size. Exogenous pressure, such as immunological microenvironment stress, acts as the “sperm,” triggering “somatic embryogenesis” by endocycling, resulting in large polyploid cancer cells (4n/pn) with a high N/C ratio. An endocycling cell goes through endomitosis or self-renewal and eventually becomes a morula-like multinucleated polyploid large cancer cell. To achieve stability, multinucleated polyploid large cancer cells become cellularized and result in a variety of undifferentiated tumors, in which pCSCs and CSCs are screened out through nuclear reprogramming. Endoreplication results in an increase in ploidy, which is referred to as a neoplastic life code. The core concept of the above hypothesis has been described previously [65]. [file 11658_2023_441_MOESM1_ESM.tif]

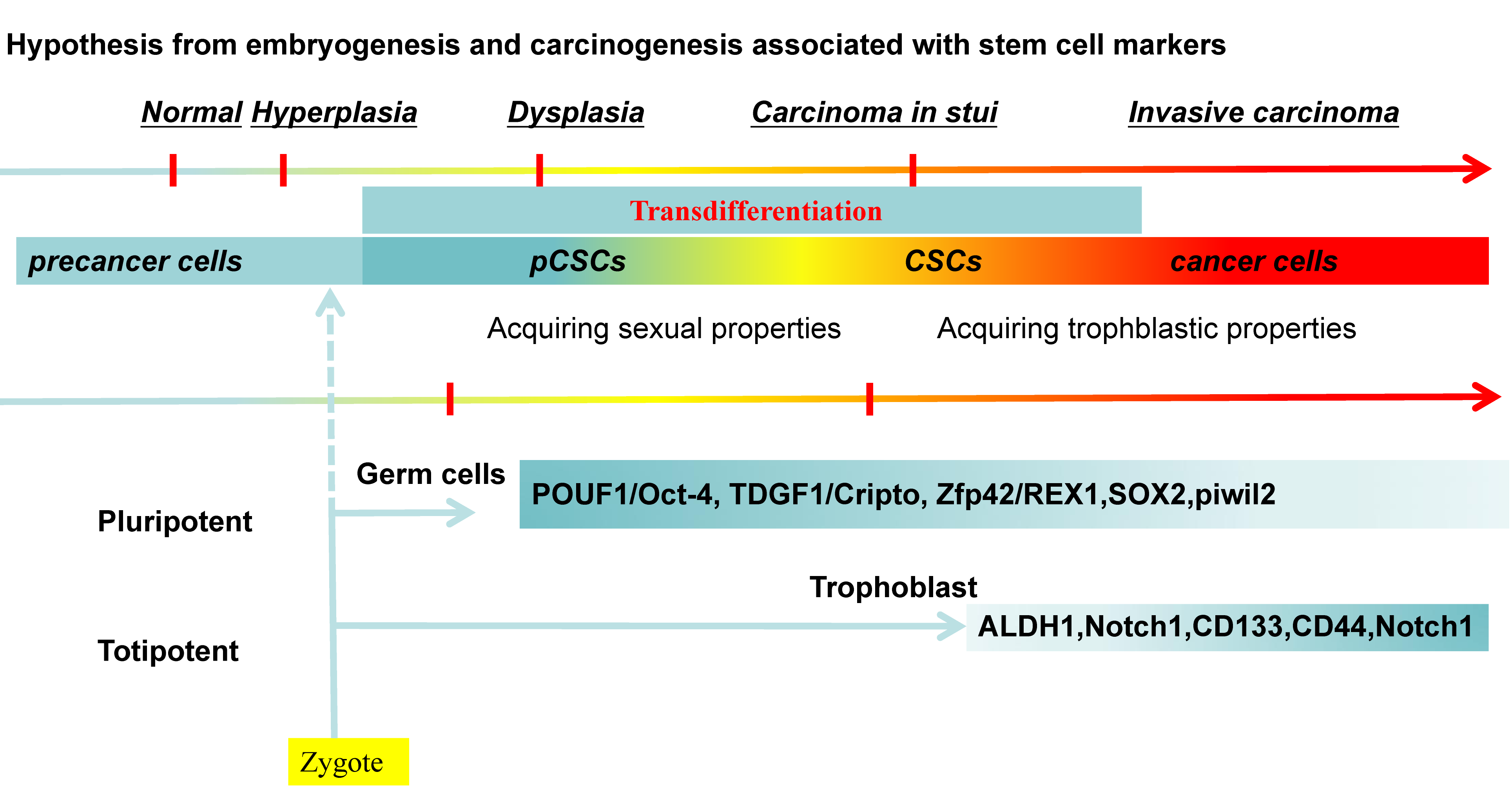

Supplement: Supplementary file 2 — Additional file 2: Figure S2. After reprogramming of typically switched off sexual and trophoblastic master genes, a precancer cell becomes malignant. Malignant cells are a phenotypic mix of primordial precancer cells with sexual-like and trophoblastic-like transdifferentiations, similar to precancerous stem cells (pCSCs) and cancer stem cells (CSCs). A relevant collateral consequence could be the expression of oncofetal biomarkers. Pouf1/Otc4, TDGF1, Zfp42/REX1, and Sox2 in pCSCs are associated with germ stem cell (GS) markers and CD24, CD44, Nestin, Sox2, Notch1, and Nanog are associated with trophoblasts. The core concept of the above hypothesis has been described previously [66]. [file 11658_2023_441_MOESM2_ESM.tif]
